# The early history of ideas on brief interventions for alcohol

**DOI:** 10.1111/add.12458

**Published:** 2014-02-12

**Authors:** Jim McCambridge, John A Cunningham

**Affiliations:** 1 Faculty of Public Health and Policy, London School of Hygiene and Tropical MedicineLondon, UK; 2 Centre for Mental Health Research, The Australian National UniversityCanberra, Australia; 3 Centre for Addiction and Mental HealthToronto, Canada

**Keywords:** Alcohol, brief intervention, controlled drinking, history, primary care, public health

## Abstract

**Aims:**

This study explores the early development of brief interventions for alcohol using a history of ideas approach with a particular focus on intervention content.

**Methods:**

The source publications of the key primary studies published from approximately 1962 to 1992 were examined, followed by a brief review of the earliest reviews in this field. These studies were placed in the context of developments in alcohol research and in public health.

**Results:**

After early pioneering work on brief interventions, further advances were not made until thinking about alcohol problems and their treatment, most notably on controlled drinking, along with wider changes in public health, created new conditions for progress. There was then a golden era of rapid advance in the late 1980s and early 1990s, when preventing the development of problem drinking became important for public health reasons, in addition to helping already problematic drinkers. Many research challenges identified at that time remain to be met. The content of brief interventions changed over the period of study, although not in ways well informed by research advances, and there were also obvious continuities, with a renewed emphasis on the facilitation of self-change being one important consequence of the development of internet applications.

**Conclusions:**

Ideas about brief interventions have changed in important ways. Brief interventions have been studied with different populations of drinkers, with aims embracing both individual and population-level perspectives, and without well-specified contents. The brief intervention field is an appropriate target for further historical investigations, which may help thinking about addressing alcohol and other problems.

## Introduction

This is a study in the history of ideas about drinking alcohol and how it may be influenced, focusing on the evolution of thinking about brief interventions. The World Health Organization (WHO) defines brief interventions as ‘practices that aim to identify a real or potential alcohol problem and motivate an individual to do something about it’ (1, p. 6). This permissive definition lacks specificity in intervention content, a characteristic also reflected in two definitions offered by Nick Heather, a key architect of contemporary thinking on brief interventions. He described brief interventions as ‘a family of interventions varying in length, structure, targets of intervention, personnel responsible for their delivery, media of communication and several other ways including their underpinning theory and intervention philosophy’ 2. The key feature of the brief intervention construct was articulated as ‘a set of principles regarding intervention (arising from the public health approach to alcohol problems)’ 3. The WHO manual for use in primary care 1 identifies four types of interventions: alcohol education for those screening negative, and simple advice, brief counselling and specialist referral for increasing levels of risk as identified by the Alcohol Use Disorders Identification Test (AUDIT) 4. We are unaware of any studies of brief interventions evaluating alcohol education for those whose drinking is not identified as risky, and thus give no further attention to this group.

To investigate the development of these ideas it is necessary to situate them in relation to prevailing ideas about alcohol problems and their treatment and contemporaneous thinking more broadly. The first brief intervention trial 5–7 was undertaken by Morris Chafetz and colleagues at Massachussets General Hospital in Boston 8, published at approximately the same time as D.L. Davies' paradigm-shifting study undertaken at the Maudsley Hospital in London, of the achievement of ‘normal drinking’ among former alcoholics 9. Davies' study is widely credited as being a seminal influence on ending abstinence as the exclusive goal of alcohol treatment, recognizing controlled drinking as being also legitimate 10.

It has been suggested previously that developments in the alcohol treatment research literature since 1940 ‘are caused less by accumulating scientific knowledge than by changes in conceptions and structurings of research and knowledge’ (11, p. 193). This may also be true of brief interventions, suggesting the possible value of a history of ideas approach to this subject. These regard ‘the primary task of the historian is to offer good representations of the past. … not primarily to offer evaluations’ 12. They have obvious limitations; they do not prioritize situating particular ideas within institutional, professional and cultural contexts, thus potentially being relatively blind to the operation of social, economic and political power when compared to detailed historiography (e.g. 13,14). They may, none the less, be useful to historical scholarship by identifying targets for further investigation.

The importance of ideas has been emphasized previously for alcohol 15. History of ideas approaches have been used within a range of disciplines relevant to addiction for various purposes, using methods including literature review. Soydan 16 uses primary and secondary data sources to construct a historical account of social work as a profession and academic discipline. Jablensky 17 questions the ongoing changes in the diagnostic systems used in psychiatry. McPhail-Bell and colleagues 18 examine background documents for the 1986 Ottawa Conference, a key event in the development of health promotion as a discipline. We seek to establish whether ideas about brief intervention have changed between 1962 and 1992 by examining texts in the research literature over this period.

## The Boston trials

The first Boston trial was the culmination of a series of studies begun in 1957 which investigated the adequacy of existing emergency room care for alcoholics 19, and included publication of preliminary findings from a subset of the study population in the year prior to the full trial results 20. Alcoholism was viewed explicitly as a behavioural problem amenable to intervention outside hospitals in the community 20. The brief intervention involved a psychiatrist and a social worker seeking to capitalize on the emergency care visit by referring the patient to out-patient alcohol treatment (a clinic had been opened in Boston earlier in the 1950s). The intervention involved ‘meeting patients initially with understanding, sympathy, and attention to expressed needs, however concrete they may be’ 21. These ideas held by the young Chafetz about the nature of alcohol problems co-existed alongside others, with an earlier report presenting a psychodynamic interpretation of alcoholism influenced heavily by Freud 22, thinking still strongly evident to at least one writer in 1961 23.

The first trial alternately assigned 200 male alcoholics to experimental and usual-care groups following diagnosis of alcoholism by the chief medical officer 8. Sixty-five brief intervention patients versus five control patients subsequently made an initial visit to the out-patient alcohol clinic, with 42 versus one completing five visits or more over a 12-month period 8. Concern about the generalizability of these data due to the very severe nature of the problems faced by the frequently homeless study population of ‘skid row alcoholics’ led to a second trial designed to assess effectiveness in a less damaged population 21. Large differences were similarly found in the second study. Chafetz went on to become the founding director of the new National Institute on Alcohol Abuse and Alcoholism in the United States at the beginning of the 1970s; however, the study of brief interventions itself was almost completely dormant during the 1960s and early 1970s 5.

## Controlled drinking: changing ideas about the nature of alcohol problems

The contemporaneous connection with the Davies study 9 was not simply coincidental. There were broader developments in thinking about alcohol problems which were to have major implications for the subsequent development of brief intervention studies, as well as for alcohol research more generally. A punitive approach criminalizing those with alcohol problems was giving way to a more liberal approach. This arose out of a concern for the effects of labelling, which was prominent in the sociology of deviance at the time 24. Attention to better understanding of the needs of drinkers became a constituent element of the progressive politics of the 1960s 25. Throughout that decade and the next the disease concept of alcoholism came to be challenged and defended robustly 26–28 in a dispute about the nature of scientific thinking on alcohol problems. There are different varieties of disease conceptions of addictions 29,30, although at its heart the disease concept involves a categorical separation of people who are unable to drink normally from those who are, making controlled drinking impossible for the former.

This was gradually challenged by explicitly behavioural conceptions of the nature of drinking problems within the treatment research community. These were already evident during the 1950s, for example in Finland, where reduced drinking was accepted as an acceptable treatment outcome measure in addition to abstinence 31. Drinking problems, even very extreme drinking leading to disease and premature death, became defined fundamentally as a behaviour, regardless of how pathological it appeared to be. Alan Marlatt and colleagues published a key study in 1973 32 that was a seminal influence in this challenge to the disease perspective. Later in the 1970s the dispute over the legitimacy of controlled drinking as a treatment goal erupted into a major scientific controversy. Detailed histories and evaluations of this controversy are available 10,33,34. The key implication for the present study is that establishing controlled drinking as a treatment goal helped to create the pre-conditions for the emergence of the modern idea of brief intervention.

In so doing, problem drinking, understood as a behaviour over which control could be exerted, invited interventions providing help. This concept fitted with the emerging epidemiological perspective, which saw alcohol consumption and problems as being distributed in degrees across the entire drinking population in a roughly normal distribution, but skewed towards the heavier end 35–37. It was also very much of its time within psychology, being part of the rise of behaviourist ideas more widely within that discipline, and in psychiatry, and the concomitant waning of influence of psychoanalytical perspectives. The ideas of Carl Rogers 38 and the humanistic psychology movement became prominent in thinking about how to help people in general with problems of any kind throughout the 1960s and 1970s. Nonetheless, humanistic and behavioural ideas had to contend with culturally powerful and thus strongly persistent ideas of character defects as they were applied to people with alcohol problems in US treatment systems and society more broadly 39.

In 1977 Edwards & Orford published a trial 40 showing equivalent outcomes for 100 married men given a single session of ‘advice’ in comparison with standard treatment of the time involving in-patient admission and extensive aftercare, including Alcoholics Anonymous (AA) facilitation. The approach used to the advice was not directed at treatment engagement, and instead directly involved wives in assisting the resolution of problems in their husbands 40. Abstinence was the treatment goal; general practitioners (GPs) were to be contacted for withdrawal medication and couples were:

… told that responsibility for attainment of the stated goals lay in their own hands rather than it being anything which could be taken over by others, and this message was given in sympathetic and constructive terms. It was explained that the patient would not be offered a further appointment at the clinic, but that someone would call each month to see the wife and collect news of progress 40.

This study arose from a sharing of ideas between clinicians such as Edwards & Orford and leading thinkers in the areas of epidemiology, alcohol policy and the natural history of addictions 41, and was recognized quickly as seminal, being discussed widely in the brief interventions literature that was to develop in the following years. Indeed, it was also very influential for alcohol treatment, leading treatments thereafter to become briefer and offered in out-patient settings.

## The primary care revolution

Also at the Maudsley hospital in London at around the same time the first primary care brief intervention trial for any health-related behaviour was being undertaken by Michael Russell and colleagues. It showed that brief GP advice to stop smoking successfully encouraged more patients to do so 42. Developments at the WHO in thinking about the role of primary care in relation to health promotion, as embodied in the Alma-Ata Declaration of 1978, provided another stimulus to interest and activity 43. These led to a major shift in thinking away from traditional approaches to alcohol treatment towards public health responses that emphasized instead ‘strategies that could be applied in primary health care settings with a minimum of time and resources’ 44. Heavier drinkers at risk of problems were identified as the key target population for ‘early’ intervention with a view to preventing later, more severe, problems 43. Brief interventions conceived in these ways served both individual- and population-level prevention purposes.

Tom Babor was a key figure in the major WHO brief interventions international collaborative project that developed through the 1980s and produced the AUDIT 45, and subsequently involved a seminal trial in 10 diverse countries 44,46,47. His name is to be found as first author on many of the key WHO publications, including the first review paper on brief interventions in primary care, published in 1986 as part of this initiative 43. This discussed the rationale, context, evidence and methods of brief intervention in primary care; yet only findings from two alcohol trials were able to be described at that time. Neither of these was in primary care, and both were restricted to men only 43.

The Malmo study by Kristenson and colleagues 48 resulted from a large city-wide screening programme undertaken by the university preventive medicine department. Intervention among problem drinkers in the top decile of the gamma glutamyl transferase (GGT) distribution involved successive contacts with hazardous drinkers by both doctor and nurse and, like the studies by Chafetz and colleagues, the results were remarkable by today's standards. Large effects on alcohol and mental health outcomes were accompanied by impacts on health services utilization, employment and mortality 48. Unlike most subsequent brief interventions, ongoing laboratory test results were very prominent:

Patients were offered continuing follow-up with consultations with the same physician every third month and monthly GGT tests and reinforcing contacts with the same nurse. Counselling was focused on living habits. A treatment goal of moderate drinking rather than abstinence was agreed upon. The individuals were considered to have their weaknesses and frequently exhibited slight contradictions in personal contact situations, but this was not regarded as disease. The subjects were offered support and encouragement in their efforts to change drinking habits but were given full responsibility for the outcome of their participation. Moderate drinking was tolerated when necessary as long as GGT values did not rise 48.

The other trial, by Jonathan Chick and colleagues, took place in the general hospital setting in Edinburgh 49, targeting problem drinkers 49. After assessment, the brief intervention comprised:

counselling from the nurse. The session lasted up to 60 minutes, during which the nurse gave the patient a specially prepared booklet and engaged him in a discussion on his lifestyle and health, which helped him to weigh up the drawbacks of his pattern of drinking and to come to a decision about his future consumption. The objective was to help the patient towards problem free drinking, though abstinence was the agreed goal for some 49.

## A golden age?

These findings, along with the growing international collaboration fostered by the WHO project, inspired a rapid upsurge in research activity on brief interventions. The research advances made in the period after the 1986 review were summarized and analysed in a review by Bien and colleagues in 1993 5. With the exception of the report of the AUDIT development, this is the most-cited brief intervention study of any kind, cited more often than any primary trial (more than 780 times in October 2013), and much more often than the next most-cited review (50 cited approximately 455 times). Bien and colleagues summarized evidence in three areas; referral to and within treatment services; those delivered in other health services; and general population-targeted media-recruited studies (e.g. advertisements in newspapers) 5. Forty-four controlled trials were included. The first primary care-based trial was started around 1982, although not published until 1987 51. The contents of brief interventions included manuals/self-help, feedback and advice, brief counselling and referral. Although most interventions were single-session, primary care-based studies had not yet become especially prominent, and the evidence supported all evaluated forms of intervention 5.

Among the included studies were an evaluation of the Drinker's Check-Up, a two-session intervention designed to implement the counselling style of motivational interviewing (MI) in a brief intervention format, seeking to attract and engage problem drinkers in non-stigmatized assessment and feedback 52. The first author, Bill Miller, was the original author of MI 53 and of other included studies, and a co-author of the review itself. The first edition of the textbook on this counselling style 54 included content on brief interventions, including a conversational topic-based approach by co-author Stephen Rollnick 55, who was later to become a major influence on brief interventions. Subsequently, Miller concentrated largely on work in treatment research. At around the same time as the development of MI, and originally associated closely with it, the transtheoretical or stages of change model of Prochaska & DiClemente 56 was to become particularly influential in smoking. Both saw behaviour change as a process in which careful nurturing of motivation was central, while the former particularly emphasized the importance of how people are spoken to and, in turn, what they say 57.

Among the key early studies (e.g. 51,58) from this period were those by Nick Heather, who was influenced by both MI and the transtheoretical model. In a 1989 paper 59, which also covered smoking, he distinguished two main types of brief intervention content, advice and what was described as condensed cognitive-behavioural therapy (CBT). This distinction pre-dated the later application of MI ideas, and he has commented more recently that the contemporary distinction to be made is between MI-based approaches and advice, with condensed CBT persisting only in self-help approaches 60.

Heather subsequently played a key role in shaping thinking about brief interventions, producing the widely accepted definitions offered earlier in elaborating their public health potential, distinguishing them from other interventions, emphasizing a key distinction between those seeking help and those not, and consequently separating treatment from non-treatment. Central to these ideas is the view that intervention should occur ‘opportunistically’ whenever contacts were made, reflecting a pragmatic orientation. This needed to be carried out widely if the public health potential of brief interventions (for example, see 61) was to be achieved. Heather himself recognized both then 2,3 and later 62 that achieving impacts on population-level health outcomes was hugely ambitious, and in all probability needed integration with other alcohol policy measures in order to fulfil this aspiration.

The conclusions reached by Bien and colleagues about what was then known and unknown 5 resonate uncomfortably well approximately 20 years later. The need to study more carefully intervention components and mechanisms of effect was emphasized 5. This was the first journal presentation of the acronym FRAMES (Feedback, Responsibility, Advice, Menu, Empathy, Self-efficacy), although it had been presented previously in the MI book 54. FRAMES was a brief summary of what was understood to be important content, and the full publication in which it was developed came afterwards 63. This was intended as a guide to further research on promising content rather than a statement of known effective intervention components. Such research was not forthcoming. Assessment reactivity was prominently flagged up as a methodological concern, with analyses showing the extent of change over time in both intervention and control groups alongside between-group differences and the use of Solomon 4-group designs 64 advocated to address this phenomenon 5. This has also not been acted upon 65,66. Uncertainty about effectiveness among more dependent drinkers was articulated in ways not dissimilar to how the lack of evidence is discussed today (see 67,68).

Perhaps the publication of the WHO cross-national efficacy trial in 1992 44 or the review by Bien and colleagues published the following year 5 may be taken to represent the end of a golden age of a decade or so in which a solid foundation for future effectiveness and implementation study was formed. Although advances have continued to be made in the 20 years since, we suggest that this was the period in which a truly new paradigm had been forged.

## Contemporary relevance

The perspective outlined here obviously extends the horizons of the brief interventions construct well beyond conversations comprising time-limited advice and/or counselling, the two main brief interventions applications 60, and also well beyond primary care, the setting in which the evidence has been developed most assiduously in the intervening years 60. So, too, do the early studies. As brief interventions have, in the decades since, moved slowly from simply being objects of research scrutiny to also become large-scale national implementation programmes in primary care and other health settings 62, there has been a need to define precisely what it is that managers and practitioners are being asked to do that will implement public health principles. Hence, the idea of brief intervention has been taken increasingly to be synonymous with advice, or sometimes also including counselling, although delivery in routine practice in services is usually very brief, or ‘minimal’, to use another term introduced by Heather 69, often taking much less than 5 minutes 70,71. Despite the compilation of an evidence-base in primary care over approximately 25 years, there remains a lack of clarity about the contents of brief interventions 72 and the extent to which existing trials can be interpreted as efficacy or effectiveness studies with important implications for the generalizability of these data 60,70,73.

A further era of rapid progress in research activity on brief interventions, perhaps comparable to that of the late 1980s and early 1990s, is now occurring. This is particularly true in relation to implementation research on large programmes within health services, in some of which alcohol interventions are integrated within other health-care needs, and referral is again becoming more prominent in brief interventions 70,71,74–81. The aspiration to intervene early to prevent problems developing in the first place, or becoming worse, is enduringly appealing for many reasons, particularly as young people are targeted more widely as awareness grows of the need to do so 82–84. Brief interventions delivered by computers and the internet, for whom university students are prominent in the emerging literature 85–90, is another area which has seen major upsurges in research activity. Internet interventions herald new possibilities for developing the content of brief interventions and potentially pose something of an identity crisis for brief interventions. Because extensive and recurrent reach is possible, unmediated by human contact, this means that interventions arising from the public health understanding of alcohol problems no longer need to be brief (for example, see 91). Although comparative studies exist 92, studies of the integration of brief conversations and information technology have not, however, advanced far. A further theme running through recent developments is the need to balance multiple intervention possibilities for a range of different behaviours or problems in a complementary package (for example 93). One of the most interesting consequences of the development of the internet for ideas about brief interventions and their content has been the renaissance of an overt emphasis on the facilitation of self-change 94.

Brief interventions for alcohol make sense because encouraging people to engage pro-actively in assisted reflection on this behaviour, whether or not they have problems, is itself a key public health challenge in the general population. For other behavioural problems, such as smoking cessation, this probably matters less than more technique- and content-focused efforts to help those who have already decided to change, and briefer interventions are less effective than more extended interventions 95. This certainly does not mean that brief intervention content does not matter for alcohol, rather that it matters differently, and the slow pace of progress over the longer term needs to be rectified urgently for this literature to develop further 72. With the exception of MI, it is not obvious that other psychological treatment approaches have been influential in the development of brief interventions over time, although this may be changing 96. Where problems are more obvious, it may be that the idea of brief intervention is less attractive, whereas the converse may be true for efforts to promote physical activity, sexual health and mental health, and to reduce risks associated with overeating or drug use. The WHO continues to be a key stimulus to work on brief interventions, sponsoring the International Network on Brief Interventions for Alcohol and Other Drugs (INEBRIA) (http://www.inebria.net), the contemporary international network in this area, which has recently extended its focus to include drug use.

## Conclusions

Ideas about brief interventions changed in important ways between 1962 and 1992. The populations among whom they were evaluated broadened from existing problem drinkers to also include heavy drinkers who may later develop problems, making preventing rather than treating problem drinking more important aims. This reflected the influence of a public health conception of the possible value of brief interventions. Ideas about brief interventions have not been insulated from developments in thinking about the nature of alcohol problems. Pressures on time in primary care and other busy settings make the idea of brief intervention enduringly attractive and compel innovations in thinking about content 70,85. Changing ideas about brief intervention content have not obviously been shaped strongly by research advances, as appears to also be the case for alcohol treatment 11. Brief interventions have been more focused on making and taking opportunities for interventions than well-specified activities with distinct characteristics. The brief intervention field is an appropriate target for further historical investigations that place these changes in ideas into their wider contexts. Such studies can inform new thinking about alcohol problems more broadly. There is a rich heritage to draw upon in developing and targeting appropriate content, whatever it is, whenever and however it is delivered, to meet more effectively the needs of people struggling to control their drinking, or who may otherwise benefit from thinking afresh about a difficult issue. We should draw upon it.
